# Phylogenetic Portrait of the *Saccharomyces cerevisiae* Functional Genome

**DOI:** 10.1534/g3.113.006585

**Published:** 2013-08-01

**Authors:** Patrick A. Gibney, Mark J. Hickman, Patrick H. Bradley, John C. Matese, David Botstein

**Affiliations:** * The Lewis-Sigler Institute for Integrative Genomics, Princeton University, Princeton, New Jersey 08544; † The Department of Molecular Biology, Princeton University, Princeton, New Jersey 08544; ‡ Department of Chemistry and Biochemistry, Rowan University, Glassboro, New Jersey 08028

**Keywords:** yeast, evolution, phylogeny, orthology, genome

## Abstract

The genome of budding yeast (*Saccharomyces cerevisiae*) contains approximately 5800 protein-encoding genes, the majority of which are associated with some known biological function. Yet the extent of amino acid sequence conservation of these genes over all phyla has only been partially examined. Here we provide a more comprehensive overview and visualization of the conservation of yeast genes and a means for browsing and exploring the data in detail, down to the individual yeast gene, at http://yeast-phylogroups.princeton.edu. We used data from the OrthoMCL database, which has defined orthologs from approximately 150 completely sequenced genomes, including diverse representatives of the archeal, bacterial, and eukaryotic domains. By clustering genes based on similar patterns of conservation, we organized and visualized all the protein-encoding genes in yeast as a single heat map. Most genes fall into one of eight major clusters, called “phylogroups.” Gene ontology analysis of the phylogroups revealed that they were associated with specific, distinct trends in gene function, generalizations likely to be of interest to a wide range of biologists.

The genome of the model eukaryote *Saccharomyces cerevisiae* was first sequenced in 1996, providing the first glimpse of what turned out to be a highly conserved eukaryotic gene set (Goffeau *et al.* 1996). To compare evolutionary and functional relationships between genes of different species, it is useful to compare orthologs. Orthologs are genes whose sequences most closely resemble that of a common ancestor but have since diverged through speciation. Orthologs often retain the same function in the two diverged species. For example, the catalytic subunit of the replicative DNA polymerase is encoded by orthologs: *POL2* in *S. cerevisiae* and *POLE* in human (D’Urso and Nurse 1997). Knowing all of the orthologs of a gene is useful in many ways. First, if the function of a gene is known in one species, the gene function(s) of orthologs in other species can be inferred with some confidence, although not with certainty. Second, ortholog analysis can illustrate the contexts in which the gene is important. An ortholog that is conserved in all organisms might well be critical for basic cell biology, whereas a gene conserved in only fungi may encode a protein that is required for a fungal-specific process, like spore formation. Third, knowing the species in which a gene is conserved has medical applications. For example, if a gene is specific to fungi, the encoded protein could represent an attractive target for antifungal drugs.

An early attempt to compare the protein-encoding gene sets of four species, namely *Escherichia coli*, *S. cerevisiae*, *Caenorhabditis elegans*, and *Homo sapiens* revealed a striking number of similar proteins among these species (*C. elegans* Sequencing Consortium 1998). Because the genome sequences were then still not completely known, these authors could not determine the orthology of full-length proteins but instead relied on proteins having similar domains (Sonnhammer and Durbin 1997). Another early study compared the complete gene sets of *S. cerevisiae* and *C. elegans* (Chervitz *et al.* 1998). This study inferred orthology by the method of reciprocal BLAST, whereby the two genes were assumed to be orthologs if each was the best hit when the other was used as a query in a BLAST search of the other genome, with a set minimum significance score (Altschul and Gish 1996). Using this reciprocal BLAST approximation, the authors were able to determine that 40% of *S. cerevisiae* genes and 20% of *C. elegans* genes are orthologous and that these proteins carry out a set of core biological processes (intermediary metabolism, DNA/RNA metabolism, protein folding, trafficking, and degradation; Chervitz *et al.* 1998). The major limitation of this study was that yeast and worm were the only complete eukaryotic genome sequences available at the time.

Today, hundreds of diverse genomes have been sequenced. However, not all these genomes have been completely annotated, and each “orthogroup” (*i.e.*, the group of genes from different species that are all orthologs of each other) has yet to be fully defined. However, one major effort, OrthoMCL, has undertaken to identify all of the orthogroups in about 150 fully sequenced and annotated genomes (Chen *et al.* 2006; Li *et al.* 2003). OrthoMCL also begins with reciprocal BLAST, as described previously, but uses additional steps to prevent closely related paralogs (genes duplicated within a species that remain highly similar) from forming separate orthogroups. The result is a collection with thousands of orthogroups, each containing a group of orthologs and paralogs from ~150 different species.

In this study, we used the data from OrthoMCL to globally examine and visualize the conservation of *S. cerevisiae* genes across the same 150 species, which represent a wide range of species spanning the archaea, bacteria, and eukaryota. A clustered global heat map was constructed that makes it easy to assess the pattern of conservation for each *S. cerevisiae* gene over all the species. These patterns defined eight major clusters of genes, which we call “phylogroups.” The phylogroups were queried for enrichment of gene ontology (GO) terms to reveal any functional logic underlying the patterns of conservation. This analysis produced a phylogenetic portrait of the *S. cerevisiae* genome, showing gene conservation and functional cohesiveness across the domains of life. Accompanying this study is an interactive website that allows users to explore the data in greater detail, including the ability to search yeast phylogroups for individual genes of interest (http://yeast-phylogroups.princeton.edu).

## Materials and Methods

### Acquisition and processing of ortholog and yeast genome data

Data defining orthologs to yeast genes were downloaded from OrthoMCL (www.orthomcl.org). Data regarding yeast gene annotation were downloaded from Saccharomyces Genome Database (www.yeastgenome.org). Processing and combining these data are described in more detail in Supporting Information, File S1 and File S2. Data were organized and processed using a combination of Microsoft Excel, and R (www.r-project.org). Data were visualized using R or MultiExperiment Viewer (MeV_4_7, version 10.2; www.tm4.org/mev/).

### Arranging yeast genes into phylogroups

**Figure 1:**
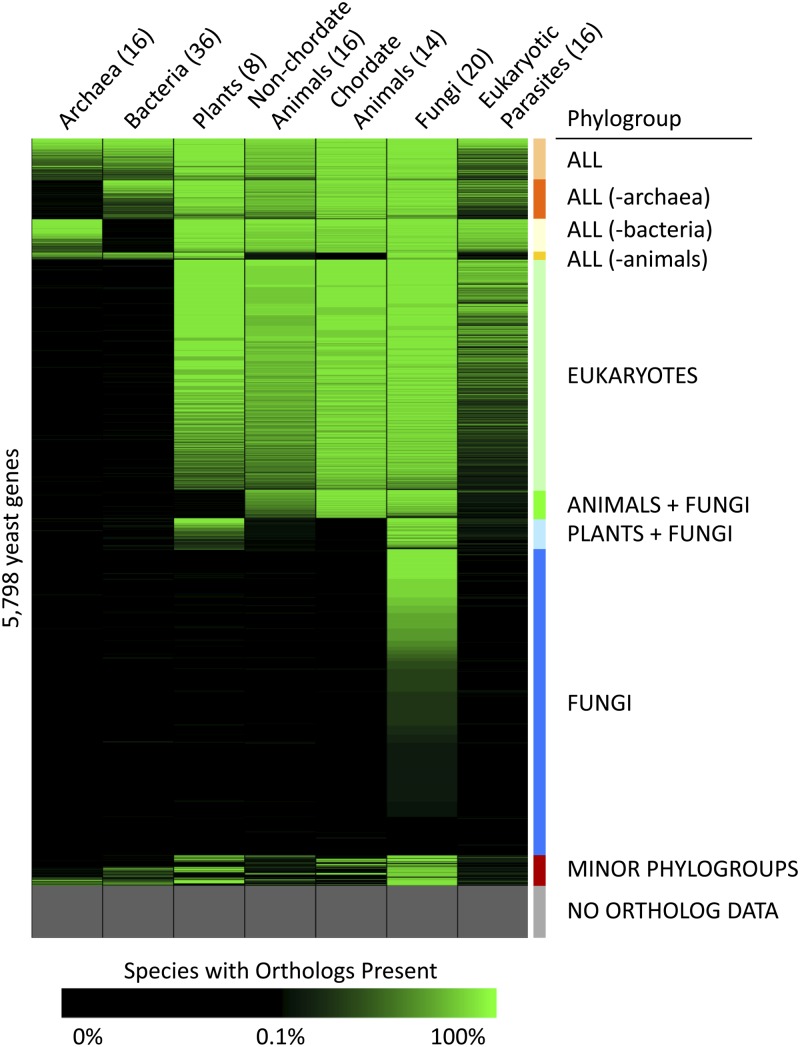
The *S. cerevisiae* genome grouped into distinct phylogenetic categories based on genetic conservation. Green intensity indicates percent of species in a specified taxonomic category (listed along the top with number of species analyzed in each category shown in parentheses) with orthologs to the *S. cerevisiae* genes (along the y-axis) (see legend below for color-to-percent conversion). Genes were ordered as described in the section *Materials and Methods*. Distinct phylogenetic categories, which we call phylogroups, are listed descriptively to the right of the color-map. The “minor phylogroups” are examined in finer detail in Figure S2. The “No Ortholog Data” phylogroup refers to a set of yeast genes that were not curated by the OrthoMCL database and so were not analyzed in this manuscript.

Yeast genes were ordered into phylogroups on the basis of shared patterns of conservation among six taxonomic groups (archaea, bacteria, plants, nonchordate animals, chordate animals, and fungi) (Figure 1). A threshold of 20% was chosen to ensure that at least two organisms of a given taxonomic category contained yeast orthologs for including a gene into a phylogroup. Phylogroups containing less than 50 yeast genes were placed together in a group called “Minor phylogroups” (the range of yeast genes in each minor cluster phylogroup varies from 1 to 32). Genes within each phylogroup were sorted according to the total number of species in which the gene is conserved. For the purposes of defining and ordering genes within phylogroups, eukaryotic parasites were not considered due to the phenomenon of parasitic organisms losing various genes depending upon their type of host parasitism. The data for eukaryotic parasites subsequently were added to the data set after all group definition and ordering was completed. Different sets of genes (essential, uncharacterized, etc.) were assessed for distribution among phylogroups (Figure S4). A number of other methods were tested for defining phylogroups, including hierarchical clustering methods (for example, Euclidian distance-based clustering of data binarized around the 0.2 threshold). These methods lead to similarly defined phylogroups (Figure S5).

### GO-Slim analysis

**Figure 2:**
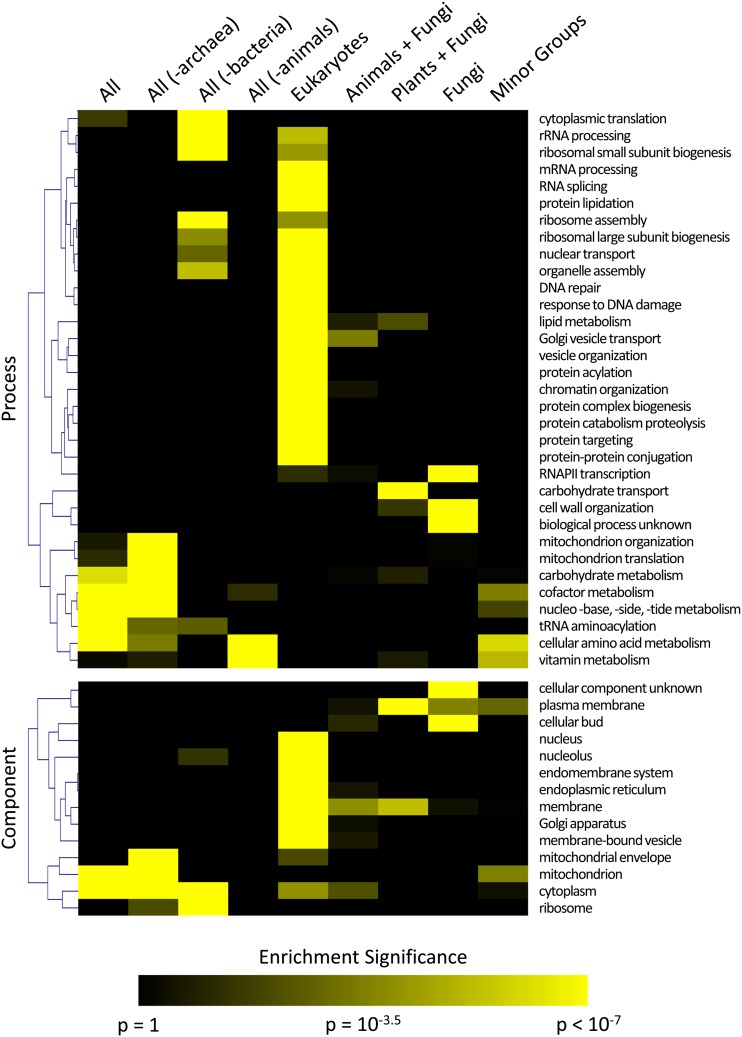
GO term enrichment of phylogroups. GO-Slim Mapper was used to identify GO terms that are enriched in each phylogroup. The most significant results are presented in a color-map with yellow intensity corresponding to significance of enrichment (see legend; the color intensity scale was defined using our significance threshold of *P* < 10^−7^). Phylogroups analyzed are listed across the top of the color-map. GO-Slim Categories are shown in the indicated order: process (top), component (bottom). The data for GO-Slim Category: function is presented in Figure S3.

GO analysis was performed using the GO-Slim Mapper tool implemented in the Saccharomyces Genome Database (http://yeastgenome.org/cgi-bin/GO/goSlimMapper.pl). GO-Slim Mapper was used rather than standard GO Term finder because of the smaller, less redundant number of ontology terms used. *P*-values were calculated using the cumulative hypergeometric distribution and then corrected for multiple-hypothesis testing via use of the Benjamini-Yekutieli method (Benjamini and Yekutieli 2001). For display of GO term enrichment in Figure 2 and Figure S3, GO terms were only included if at least one phylogroup had a significant enrichment (*P*-value of at least 1 × 10^−7^). The full set of GO Slim results is available for download from http://yeast-phylogroups.princeton.edu. Each table of GO terms was hierarchically clustered using Kendall’s tau (a rank-order based statistic) as the clustering metric and average linkage as the linkage method.

## Results

By combining publicly available data for yeast gene annotation (www.yeastgenome.org) and for ortholog prediction (www.orthomcl.org), we created a clustered map of the yeast genome based on gene conservation. After organizing the data so that yeast genes with similar patterns of gene conservation were clustered together, it became clear that we could simplify the display by amalgamating the 126 individual species into seven taxonomic groups (archaea, bacteria, nonchordate animals, chordates, eukaryotic parasites, fungi, and plants) and indicating the presence of an ortholog to a yeast gene by the intensity of color (Figure S1 and Figure 1).

The yeast genes in Figure 1 fall into distinct phylogenetic categories, hereafter called “phylogroups.” Among these are eight major phylogroups: “All” (genes well-conserved in all taxonomic categories), “All (except archaea)” (genes well conserved in all taxonomic categories except archaea), “All (except bacteria)” (genes well conserved in all taxonomic categories except bacteria), “All (except animals)” (genes well conserved in all taxonomic categories except animals), “Eukaryotes” (genes well conserved in all eukaryotic taxonomic categories), “Animals and Fungi” (genes well conserved in animals and fungi, but not the other taxonomic categories), “Plants and Fungi” (genes well conserved in plants and fungi, but not the other taxonomic categories), and “Fungi” (genes well conserved in fungi). The remaining two groups are marked in Figure 1 as “Minor Phylogroups” (containing 19 phylogroups with <50 yeast genes each; see Figure S2), or “No Ortholog Data” (yeast genes that were not curated into the OrthoMCL database).

### Genes in phylogroups exhibit functional cohesiveness

To assess biological roles enriched within phylogroups, the yeast gene complement from each group were examined using GO-Slim Mapper (Ashburner *et al.* 2000). The results of the “Process,” “Component,” and “Function” GO-term analyses are presented as color maps with color intensity representing the degree of statistical significance for the enrichment (Figure 2 and Figure S3).

### Phylogroup: All

This phylogroup consists of 305 yeast genes that are well-conserved in all taxonomic categories. This set of genes is significantly enriched for core metabolic GO processes (*e.g.*, carbohydrate, cofactor, amino acid, and nucleic acid metabolism), generation of precursor metabolites, tRNA aminoacylation, and response to oxidative stress. GO functional enrichments include hydrolase, ligase, oxidoreductase, and lyase activities. Included in this phylogroup are the genes responsible for pyrimidine biosynthesis (*URA1*, *URA2*, *URA3*, *URA5*, *URA7*, *URA8*, and *URA10*), purine biosynthesis (ADE4, ADE6, ADE5,7, ADE16, and ADE17), central carbon metabolism (*PGI1*, *PGK1*, *ENO1*, *ENO2*, *PYK2/CDC19*, *PYC1*, *PYC2*, *ACO1*, *ACO2*, *IDH2*, *LPD1*, *LSC1*, *LSC2*, *FUM1*, *SDH1*, *SDH2*), and some amino acid biosynthesis (aspartate, asparagine, cysteine, serine, glycine, glutamate: *ASN1*, *ASN2*, *ASP1*, *CYS3*, *CYS4*, *SER2*, *SER3*, *SER33*, *AGX1*, *GDH1*, and *GDH3*). These results suggest that this subset of yeast genes represents core metabolic processes common to all organisms on earth.

### Phylogroup: all (except archaea)

This phylogroup consists of 282 yeast genes that are well-conserved in all taxonomic categories except the archaea. This set of genes is significantly enriched for metabolic and mitochondrial processes, ATPase activity, oxidoreductase activity, and cytoplasmic/mitochondrial cellular components. These results suggest that this set of yeast genes also represents core metabolic processes, although likely with a stronger focus on functions performed in mitochondria. These include genes responsible for the production of the mitochondrial ribosome (*MRPL* and *MRPS* genes), and for some steps of carbon metabolism (*PFK1*, *PFK2*, *TPI1*, *TDH1*, *TDH2*, *TDH3*, *GPM1*, *KGD1*, *KGD2*). Notably, although the biological process and component terms are similar to the previous phylogroup (which includes the archaea), some of the enzymatic activities are different, indicating that somewhat-different patterns of metabolic reactions are represented in this group (Figure S3). Indeed, phosphofructokinase from some archaea is known to be biochemically divergent, relying on ADP as a cofactor instead of ATP, and many archaea appear to lack GAPDH (*i.e.*, *TDH1*, *TDH2*, *TDH3*) entirely, substituting an archaeal-specific enzyme that reduces glyceraldehyde using ferredoxin instead of NADH (Verhees *et al.* 2003).

### Phylogroup: all (except bacteria)

This phylogroup consists of 242 yeast genes that are well-conserved in all taxonomic categories except the bacteria. This set of genes is significantly enriched for processes, functions, and components involved in translation and, to a lesser degree, transcription. The main feature distinguishing this set of yeast genes reflects the well-documented differences between ribosomes and other translation machinery in bacteria, and the cytoplasmic translation machinery in eukaryotes and archaea (Allen and Frank 2007). This phylogroup includes genes that produce cytoplasmic ribosomes and regulate translation (*RPL* and *RPS* genes, *eIF* and *eEF* genes) and RNA polymerase II (*RPB2*, *RPB3*, *RPB5*, *RPB7*, *RPB10*, *RPB11*).

### Phylogroup: all (except animals)

This phylogroup consists of 51 yeast genes that are well-conserved in all taxonomic categories except animals. This set of genes is significantly enriched for only two processes: vitamin and amino acid metabolism. The main features of this group reflect the fact that animals generally show nutritional requirements for a subset of essential amino acids and vitamins because they have lost, over evolutionary time, the ability to synthesize them *de novo*. This phylogroup includes genes that are responsible for the production of vitamins (*BIO3*, *RIB1*, *RIB3*, *RIB4*, *RIB7*, *THI7*, *THI20*, *THI21*, *THI22*) and some of the “essential” amino acids, namely methionine, threonine, and tryptophan (*MET1*, *MET3*, *MET6*, *MET16*, *MET17*, *HOM2*, *HOM3*, *HOM6*, *THR1*, *TRP2*, *TRP3*, *TRP4*, and *TRP5*).

### Phylogroup: eukaryotes

This large phylogroups consist of 1671 yeast genes that are well-conserved in eukaryotes but not bacteria or archaea. Among this set of genes, many biological processes are significantly enriched. These include organelle organization, chromatin localization, trafficking, cell cycle, protein phosphorylation, and posttranslational protein modification/degradation, among others. Cellular components enriched in this set include cytoplasm, nucleus, endoplasmic reticulum, Golgi apparatus, and vesicles. These results suggest that this phylogroup includes genes involved in a large number of cellular processes that might be thought of as defining the core eukaryotic-specific genome, including all the genes encoding eukaryote-specific functions. These include those that govern the characteristic eukaryotic cell cycle and biogenesis of the organelles (*e.g.*, nucleus, endoplasmic reticulum, Golgi apparatus, etc.). Some genes found in this phylogroup include those that encode histone subunits (*HHT1*, *HHT2*, *HTB1*, *HTB2*, *HHF1*, *HHF2*, *HTA1*, *HTA2*), tubulin subunits (*TUB1*, *TUB2*, *TUB3*), and cyclins (*CLB1*, *CLB2*, *CLB3*, *CLB4*, *CLB6*). Despite the bacterial origin of mitochondria, many aspects of mitochondrial function, such as the traffic of large and small molecules between mitochondria and cytoplasm, are also encoded by the yeast genes in this phylogroup. Finally, the genes that control the characteristic mode of response to stress and maintenance of protein homeostasis (*e.g.*, translation, folding, trafficking, modification and degradation) fall into this group, including some major chaperones and co-chaperones (*SSA1*, *SSA2*, *SSA3*, *SSA4*, *SSB1*, *SSB2*, *KAR2*, *SSE1*, *SSE2*, *FES1*). Some of these proteins, especially the chaperones, are conserved throughout all domains of life. That they fall into the eukaryotic-specific phylogroup suggests that the eukaryotic versions have diverged enough to prevent ortholog identification based on amino acid sequence alone.

### Phylogroup: animals and fungi

This phylogroup consists of 205 genes well-conserved in animals and fungi but not the other taxonomic categories. Among this set of genes, the following biological processes are significantly enriched: signaling, exocytosis, endosomal transport, cytoskeletal organization, vacuole organization, protein phosphorylation, and Golgi vesicle transport. Enriched functions include kinase activity and cytoskeletal protein binding. Enriched components include cell cortex, site of polarized growth, cytoskeleton, and membrane. Among the genes found in this phylogroup are genes involved in many signaling pathways (*CLA4*, *SKM1*, *KIN3*, *RHO1*, *PKC1*, *FKH1*, *FKH2*, *SSK2*, *SSK22*) and in intracellular transport and sorting (*MYO3*, *MYO5*, *SHE4*, *TPM1*, *TPM2*, *ERV29*, *ERV41*, *VPS74*, *YCK1*, *YCK2*, *SEC16*, *AVL9*, *HSE1*). It is notable that in addition to determinants of cell polarity and cell shape in animals (*RHO1*, *CDC42*, *MYO3*, *MYO5*, etc.), this phylogroup also includes growth and metabolic regulators, notably those involving cyclic AMP (*CYR1*, *RAS1*, *RAS2*, *CDC25*, *YPK3*, etc.), which may be an indicator of important cell biology differences between this group of species and plants.

### Phylogroup: plants and fungi

This phylogroup consists of 227 genes well-conserved in plants and fungi but not the other taxonomic categories. Among this set of genes, carbohydrate transport is the only significantly enriched biological process. This set of genes includes the vast majority of the hexose transporters (*HXT1*, *HXT2*, *HXT3*, *HXT4*, *HXT5*, *HXT6*, *HXT7*, *HXT8*, *HXT9*, *HXT10*, *HXT11*, *HXT13*, *HXT15*, *HXT16*, *HXT17*). Among enriched functions are glycosyl-transferase, transmembrane transporter, and hydrolase activities mostly involved in sugar transport/metabolism and cell wall biogenesis. Membrane and plasma membrane components are the most highly enriched cellular components. These results suggest that this phylogroup reflects the functions required for making rigid carbohydrate cell walls, the major cellular feature shared by fungi and plants.

### Phylogroup: fungi

This phylogroup consists of 2,219 genes well-conserved only in fungi. The most significantly enriched processes include biological process unknown, cell wall organization/biogenesis, and transcription from RNA Polymerase II promoters. Enriched functions include molecular function unknown and a number of transcription factor functions (DNA binding, nucleic acid binding transcription factor, and protein binding transcription factor). Enriched cellular components included cellular component unknown, plasma membrane, cell wall, site of polarized growth, and cellular bud. It remains notable that surprisingly many of these genes are uncharacterized with respect to function.

### Minor phylogroups

The minor phylogroups consist of all remaining small (less than 50 genes each) phylogroups (Figure S2). In sum, the minor phylogroups contain 220 genes. Surprisingly, GO-Slim mapper identified significant GO term enrichment for the combined set of these disparate phylogroups, including ion transport and vitamin/cofactor/amino acid metabolism. Significant enrichment of metabolic processes among a phylogenetically disparate set of genes suggests that the genes within the small phylogroups may represent secondary metabolic processes that are highly specific for different phylogroups. Further analysis of genes in the minor phylogroups could reveal unexpected patterns of secondary metabolic reactions, potentially suggesting gene evolution, loss, or horizontal transfer.

### Eukaryotic parasites

The subset of yeast genes well-conserved in all phyla except for the eukaryotic parasites deserves specific mention. Orthologs of yeast genes responsible for the biosynthesis of purines (*ADE4*, *ADE6*, *ADE5,7*, *ADE16*, *ADE17*), and amino acids (*ARG1*, *ARG3*, *ARG4*, *ILV2*, *LYS12*, *ASN1*, *ASN2*) are missing from most of the eukaryotic parasite species even though they fall, otherwise, into the phylogroup “All.” This finding suggests that unlike other eukaryotes, these parasites depend absolutely on the host as a purine and amino acid source. This dependence for host biosynthetic molecules has been well documented in the case of the malarial parasite, *Plasmodium falciparum* (Gardner *et al.* 2002).

## Discussion

We performed this analysis to provide a phylogenetic-based view of the yeast genome in the expectation that this might shed light on the functional connections between yeast and a diverse set of other organisms. The analysis depends on the availability of many sequenced genomes but also on databases that provide curated information about evolutionary relationships among these sequences (OrthoMCL; Li *et al.* 2003), annotated functions of yeast genes (Saccharomyces Genome Database; Cherry *et al.* 1998), and the conservation of gene function and gene-associated processes (GO; Ashburner *et al.* 2000). This analysis revealed some familiar themes, illuminated some unexplored phenomena, and opens the door for future research possibilities, not least because it required the construction of a database in which any yeast gene can be queried for its relationship to the genes in all other major taxa. With this publication, we provide a useful tool for yeast biologists: an interactive website for examining the intersection of these data (http://yeast-phylogroups.princeton.edu). We also provide a useful approach for genome visualization and exploration based on gene conservation that can be easily applied to many different organisms.

### Phylogenetic characterization of the *S. cerevisiae* genome

Our phylogenetic analysis of the yeast genome resulted in sets of genes with distinct conservation patterns, phylogroups (Figure 1). Because the genes that constitute each group result in enrichment of coherent cellular processes, it seems likely that each phylogroup represents the genetic basis for the suite of traits that have evolved or been maintained in represented organisms (Figure 2).

Previous analysis of the yeast genome has indicated that approximately 40–50% of yeast genes are conserved in “higher” eukaryotes (Chervitz *et al.* 1998). This result is also found in the current work (Figure 1). Another observation in line with our expectations is enrichment for organellar GO terms in the eukaryote phylogroup because organelles are a defining feature of the eukaryotic lineage. It is also well-known that animals lack the ability to produce a number of essential vitamins and amino acids that must be acquired nutritionally (Berg *et al.* 2012). In the phylogroup that spans all organisms except animals, there is a significant enrichment for genes involved in vitamin and amino acid metabolism (Figure 2). In addition, the investigation of archaeal cell biology indicates that the archaeal translational machinery is more similar to that of eukaryotic cells than bacteria (Allen and Frank 2007). This also is borne out by our analysis of the “all (except bacteria)” phylogroup (Figure 2). These coarse-grained results, and some of the more specific examples given in the *Results* section, illustrate how this kind of data resource and visualization might be used.

### A tool for hypothesis generation

One application of this analysis is the generation of testable hypotheses for gene functions. The use of shared patterns of gene conservation across multiple species to perform high-throughput computational predictions of gene function has been in practice at least since 1998 and is called phylogenetic profiling (Eisen 1998; Lee *et al.* 2004; Pellegrini *et al.* 1999; Thomas *et al.* 2003). This technique has been successful in multiple instances, demonstrating the relevance of gene conservation patterns (Kensche *et al.* 2008). Approximately 1200 yeast genes (ca. 21%) remain completely unannotated in the literature or the databases and represent an interesting target for future study (Botstein and Fink 2011; Pena-Castillo and Hughes 2007). Most of the phylogroups identified here are highly enriched for a few biological processes, suggesting that the genes of unknown function within that group may be involved with one of those processes. For example, the “all (except animals)” phylogroup is highly enriched for vitamin and amino acid metabolism. This group also contains two genes of unknown function, suggesting the hypothesis that these genes are involved in vitamin or amino acid metabolism.

### Application of this approach to other organisms

The phylogenetic “portrait” of the yeast genome presented here can potentially reveal the evolutionary pressures that shaped different functional categories of the genome. It is reasonable to imagine that performing this analysis with another genome would yield a different picture. For example, if the phylogenetic “portrait” of the human genome were created, it could reveal different sets of phylogroups, perhaps representing the genetic underpinnings of phenomena such as multicellularity, development of tissue/organ systems, or even more complex cognitive phenotypes. Even now, evolutionary biologists and comparative psychologists are looking toward phylogenetic comparisons to examine cognitive traits (MacLean *et al.* 2012). Alternatively, using a different fungal organism might reveal a set of genes that have been lost in the *Saccharomyces sensu stricto* lineage, like the pirin genes (Cliften *et al.* 2006). In addition to examining eukaryotic genomes, unique and informative phylogroups could be found by using bacterial or archaeal species. It will be interesting to observe biological insights emerging from phylogenetic analyses as more whole genomes are sequenced, curated, and compared.

## Supplementary Material

Supporting Information
