# Supplementary material for: Phylogenetic Portrait of the Saccharomyces cerevisiae Functional Genome
Source: G3 (Bethesda). 2013 Aug 1;3(8):1335–40. doi: 10.1534/g3.113.006585 (PMC3737173; doi:10.1534/g3.113.006585)
Supplement: Supporting Information [file supp_3_8_1335__index.html]

Phylogenetic Portrait of the Saccharomyces cerevisiae Functional Genome — Supporting Information 

# Phylogenetic Portrait of the *Saccharomyces cerevisiae* Functional Genome

## Supporting Information for Gibney *et al.*, 2013

**Files in this Data Supplement:**

- Supporting Information - Figures S1-S5 and Files S1-S2 (PDF, 2 MB)
- Figure S1 - Expanded heat-map showing conservation of yeast genes in each of the 131 species analyzed (PDF, 687 KB)
- Figure S2 - Fine-scale analysis of Minor Phylogroups (PDF, 458 KB)
- Figure S3 - Gene Ontology (GO) functional category term enrichment of phylogroups (PDF, 173 KB)
- Figure S4 - Comparison of phylogenetic break-down amongst defined sets of yeast genes (PDF, 110 KB)
- Figure S5 - Alternative clustering approaches result in similar clusters of genes (PDF, 545 KB)
- File S1 - Supplemental Materials and Methods (PDF, 80 KB)
- File S2 - Downloadable Data (.zip, 3 MB)
