# Supplementary material for: Phylogenetic Portrait of the Saccharomyces cerevisiae Functional Genome
Source: G3 (Bethesda). 2013 Aug 1;3(8):1335–40. doi: 10.1534/g3.113.006585 (PMC3737173; doi:10.1534/g3.113.006585)
Supplement: Supporting Information [file supp_g3.113.006585_006585SI.pdf]

G3: Genes | Genomes | Genetics

Phylogenetic Portrait of the *S. cerevisiae* Functional Genome

Patrick A. Gibney[*,§], Mark J. Hickman[*,§§], Patrick H. Bradley[§], John C. Matese[§], and David Botstein[§]

§ The Lewis-Sigler Institute for Integrative Genomics and The Department of Molecular Biology, Princeton University, Princeton, NJ 08544
§§ Department of Chemistry and Biochemistry, Rowan University, Glassboro, NJ 08028

DOI: 10.1534/g3.113.006585

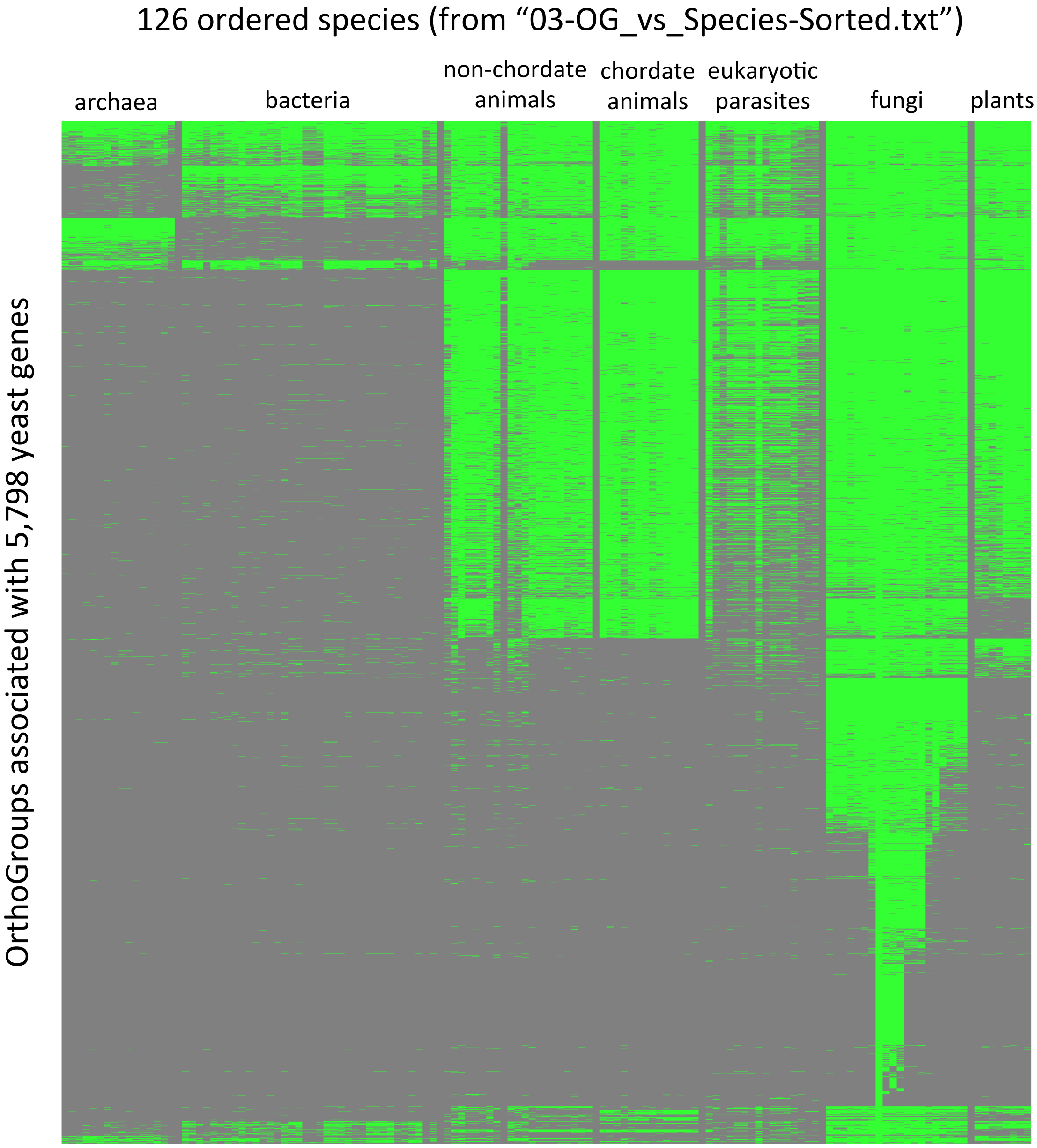

Figure S1 Expanded heat-map showing conservation of yeast genes in each of the 131 species analyzed. Binarized data representing the presence or absence of an ortholog to each protein is represented as green (presence) or grey (absence) for each of the 126 species analyzed in this manuscript (a sub-set of the species present in the OrthoMCL database). The individual species data were collapsed into taxonomic groups for Figure 1. See the Materials and Methods section for details on data binarization, species selection, and ordering of genes.

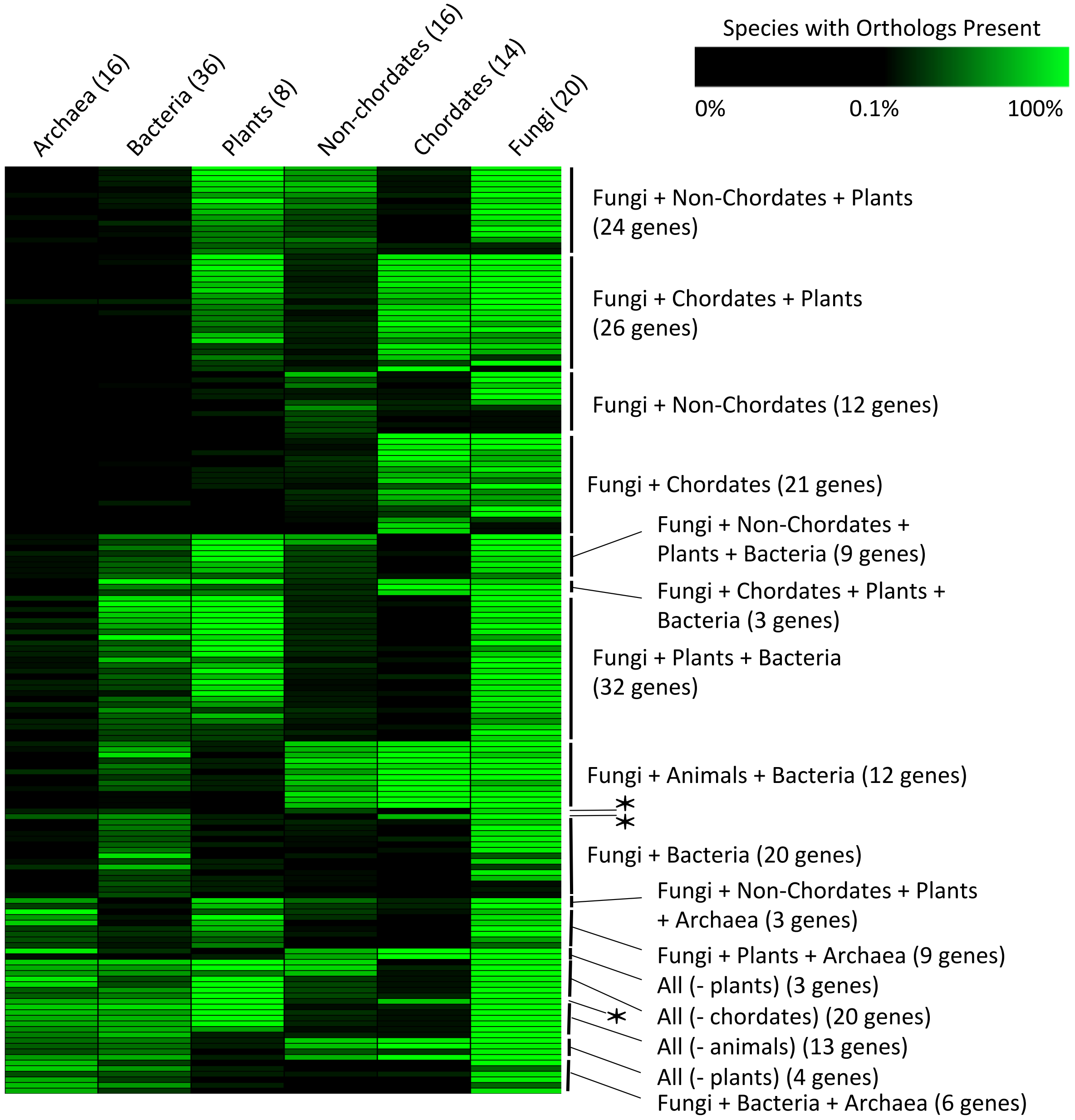

Figure S2 Fine-scale analysis of Minor Phylogroups. Expanded view of the Minor Phylogroups with included labels for rough phylogenetic categories to the right. An asterisk (*) indicates that only one gene is present with the identified phylogenetic pattern, and due to space limitations is not fully described in the phylogenetic categories to the right.

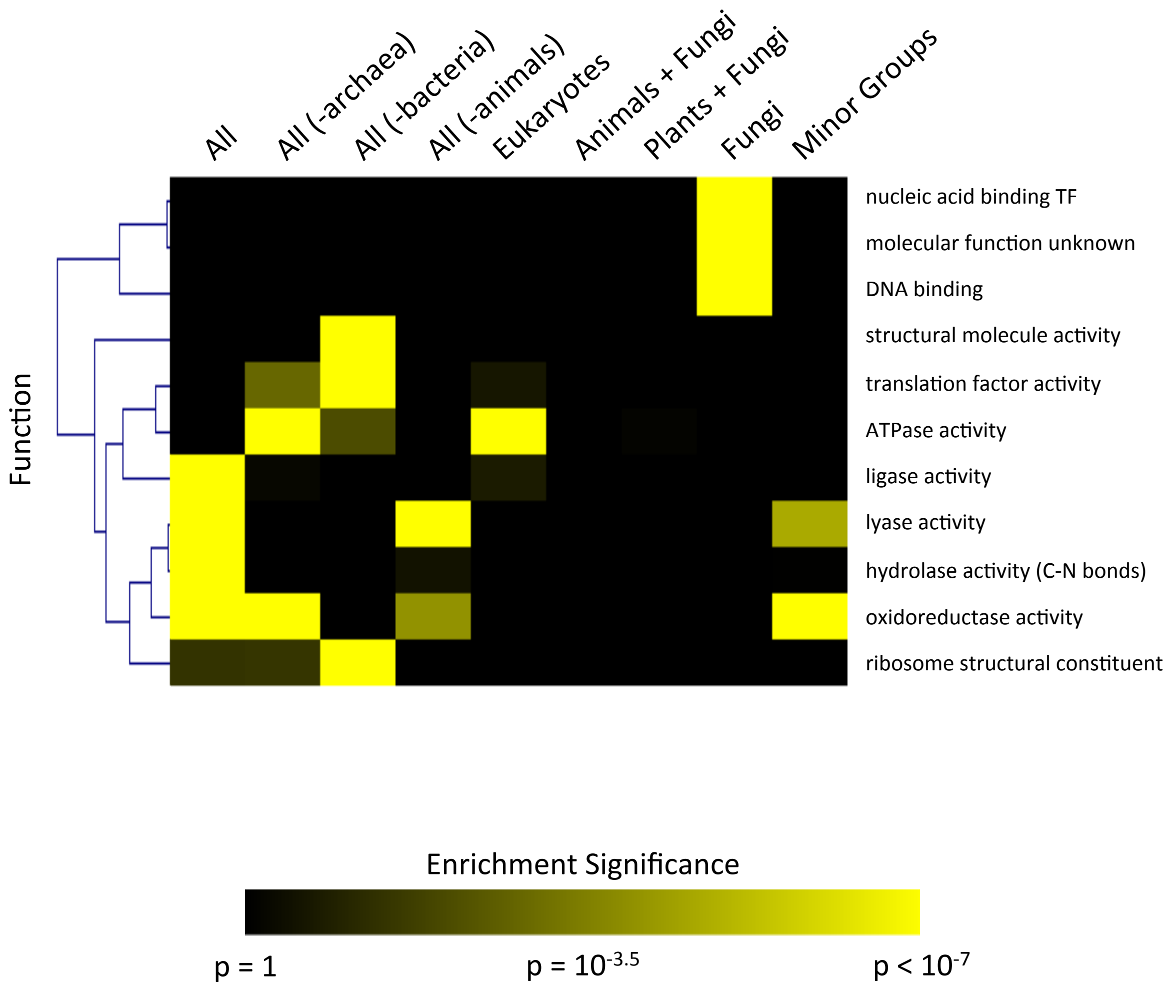

Figure S3 Gene Ontology (GO) functional category term enrichment of phylogroups. GO-Slim Mapper was used to identify GO terms that are enriched in each phylgroup. The most significant results are presented in a heat-map with yellow intensity corresponding to significance of enrichment (see legend - the color intensity scale was defined using our significance threshold of $p < 10^{-7}$). Phylogroups analyzed are listed across the top of the heat-map.

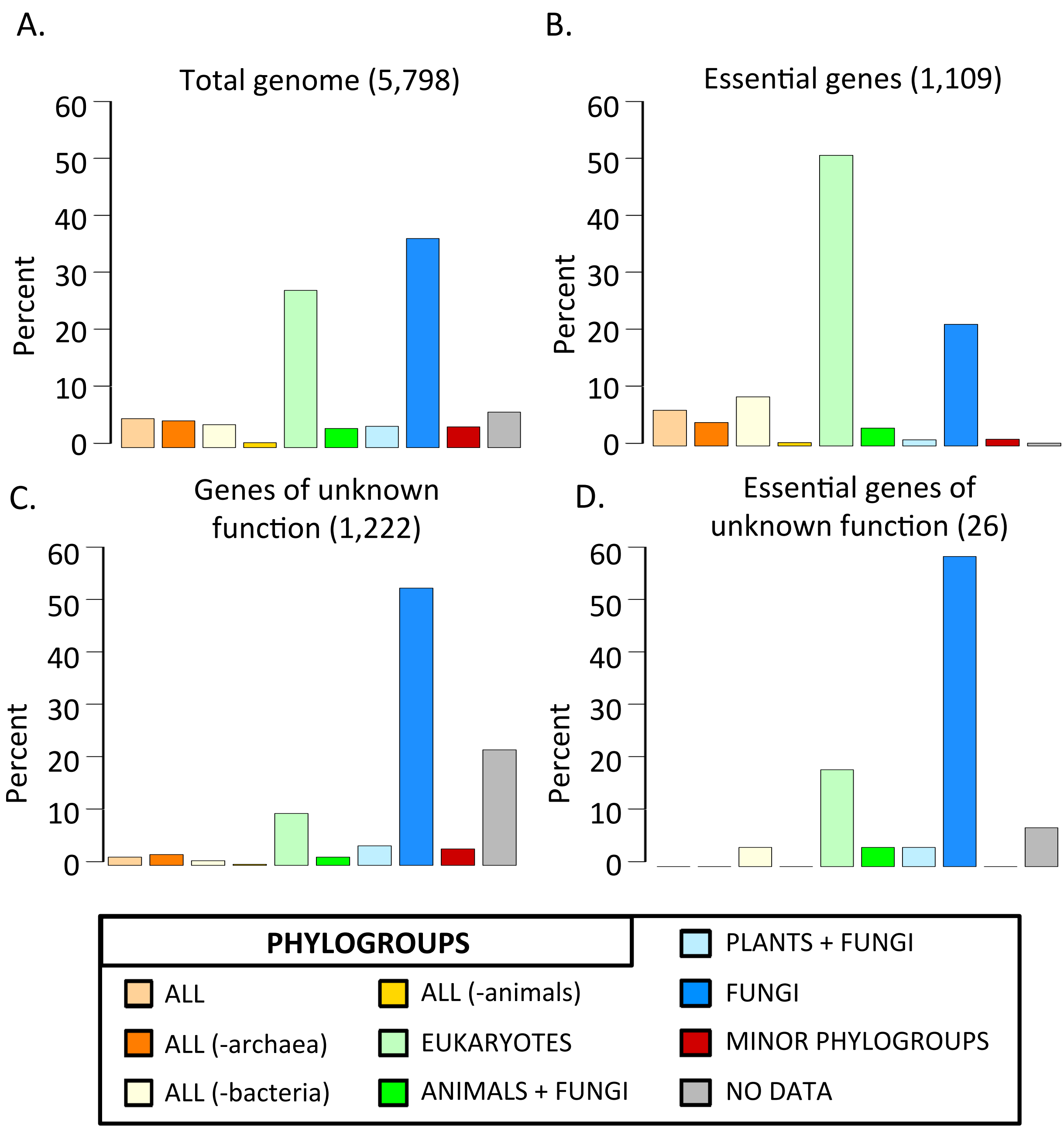

Figure S4 Comparison of phylogenetic break-down amongst defined sets of yeast genes. GO-Slim Mapper was used to identify GO terms that are enriched in each phylgourp. The most significant results are presented in a heat-map with yellow intensity corresponding to significance of enrichment (see legend - the color intensity scale was defined using our significance threshold of $p < 10^{-7}$). Phylogroups analyzed are listed across the top of the heat-map.

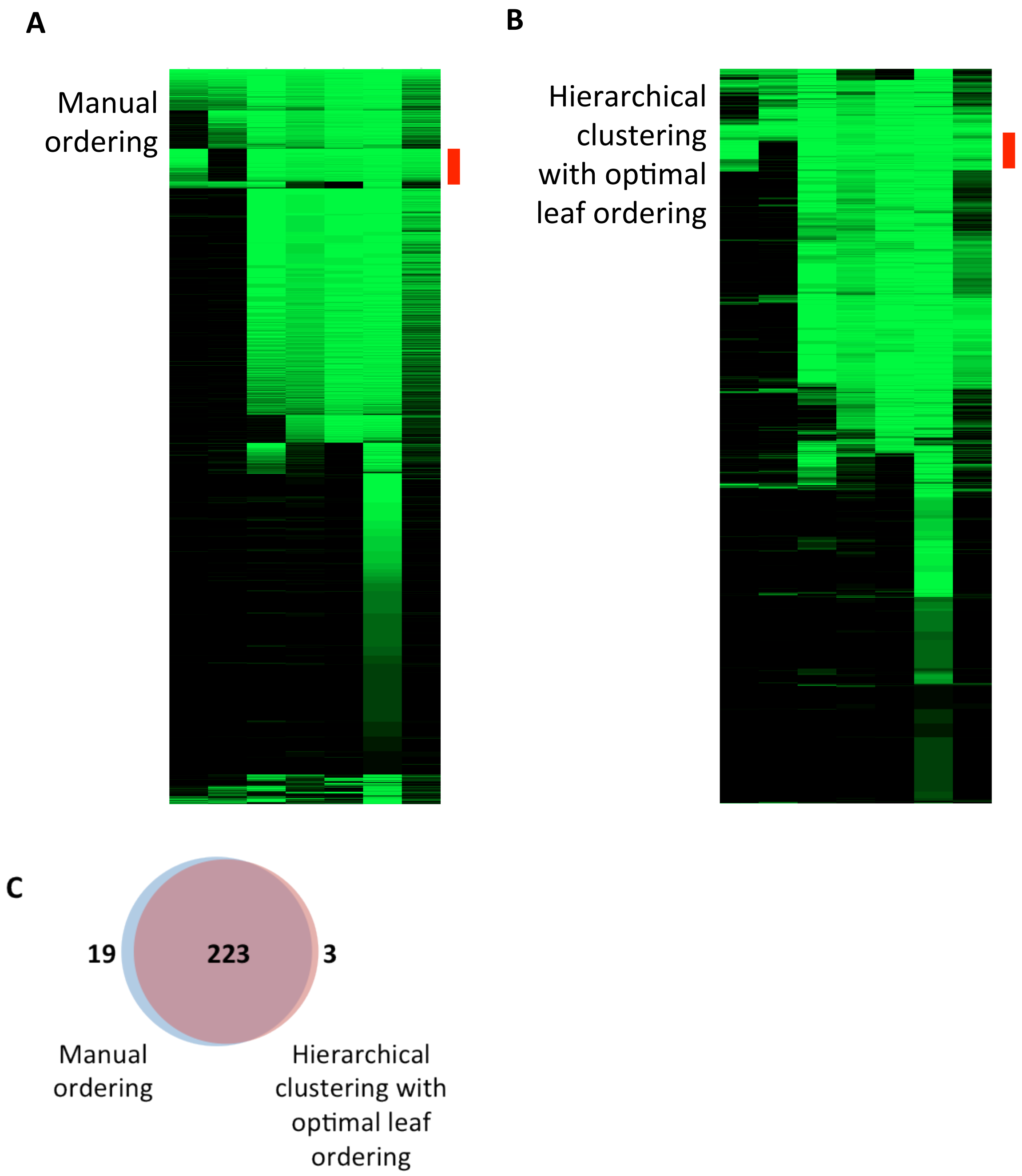
A
Manual ordering
B
Hierarchical clustering with optimal leaf ordering
C
19
223
3
Manual ordering
Hierarchical clustering with optimal leaf ordering

Figure S5 Alternative clustering approaches result in similar clusters of genes. Comparison of manual ordering (employed in this study) and hierarchical clustering with optimal leaf ordering as in Bar-Joseph, 2001 (note that in accordance with the original figure the data for panel B was binarized using the 0.2 threshold and eukaryotic parasites were not included for the clustering). (A) and (B) The column order is the same as in Figure 1. The red bar refers to the group of genes found in all species except bacteria. Note that the main difference appears to be in the scattering of genes that were placed into a group called "minor clusters" for the original figure. (C) Venn diagram showing overlap of the genes (in all species except bacteria) identified by each method. The overlap is highly significant ($p<10^{-308}$, hypergeometric distribution).

File S1

Supplemental Materials and Methods

Acquisition and processing of OrthoMCL data

Note that all files and supplementary figures are available for download at http://yeast-phylogroups.princeton.edu. Data defining orthologs for all yeast genes among the 149 other genomes curated by OrthoMCL was downloaded on July 18, 2011 (www.orthomcl.org). A file containing each yeast gene and corresponding numbers of orthologous genes from each assessed species was assembled ("01-OG_vs_Species-Full.txt"). All data regarding numbers of present orthologs per species was reduced to either "0" for no orthologs present, or "1" for at least one ortholog present ("02-OG_vs_Species-Binary.txt"). It is worth noting that a number of yeast genes (376 genes) had no orthology data in the OrthoMCL database; this is likely due to yeast gene annotation after the OrthoMCL data processing.

Because the 150 genomes curated by OrthoMCL have a handful of very closely related species (within the same genus), some species were removed to limit over-estimation of ortholog abundance within a diverse organism set simply based on overabundance of highly related species. In each case where multiple species from one genus were removed, at least one species was kept. The species removed due to genus over-representation were mostly from the "eukaryotic parasites" category: *Entamoeba histolytica* HM-1:IMSS, *Entamoeba invadens*-IP1, *Plasmodium yoelii yoelii* str. 17XNL, *Plasmodium vivax* SaI-1, *Plasmodium knowlesi* strain H, *Plasmodium chabaudi chabaudi, Plasmodium berghei* str. ANKA, *Cryptosporidium muris* RN66, *Cryptosporidium hominis* TU502, *Leishmania major* strain Friedlin, *Leishmania braziliensis, Leishmania Mexicana, Trypanosoma brucei gambiense, Trypanosoma congolense, Trypanosoma vivax, Giardia intestinalis* ATCC 50581, *Giardia lamblia* P15, and *Encephalitozoon cuniculi* GB M1 (*Magnaporthe oryzae* 70-15 was also removed because only its seventh chromosome was curated for the database, rather than its entire genome). After removing these species, a file was created with ortholog data derived from file 02 described above ("03-OG_vs_Species-Sorted.txt"). Clustering of these data revealed similar patterns of yeast gene conservation across defined taxonomic groups (Supplemental Figure 01). Therefore, ortholog data from individual species were collapsed into groups (archaea, bacteria, non-chordate animals, chordate animals, fungi, and eukaryotic parasites; see "Classification_of_OrthoMCL_Species.txt" file for full taxonomic analysis of each species). To collapse these data, the fraction of species with an ortholog to the corresponding yeast gene within a taxonomic group was calculated (the total number of species each containing at least one ortholog of a given gene was divided by the total number of species in the group)("04-OG_vs_Groups.txt").

GO-Slim analysis

Gene Ontology analysis was performed using the GO-Slim Mapper tool implemented in the Saccharomyces Genome Database (http://yeastgenome.org/cgi-bin/GO/goSlimMapper.pl). GO-Slim Mapper was used rather than standard GO Term finder due to the smaller, less redundant number of ontology terms used. Genes from each phylogroup were analyzed for enrichment/underrepresentation of three GO Slim categories (process, function, and component). P-values were calculated using the cumulative hypergeometric distribution, then corrected for multiple-hypothesis testing using the Benjamini-Yekutieli method (Benjamini and Yekutieli 2001). For display of GO term enrichment in Figure 02 and Supplemental Figure 03, GO terms were only included if at least one phylogroup had a significant enrichment (p-value of at least 1×10^{-7}). The full set of GO Slim results is available for download from http://yeast-phylogroups.princeton.edu. Each table of GO terms was hierarchically clustered using Kendall's Tau (a rank-order based statistic) as the clustering metric. Average linkage was used as the linkage method. GO term leaf order was also optimized. MultiExperiment Viewer was used to perform clustering.

Yeast genome feature and phenotype information

Files containing gene feature information and gene-associated phenotype information were downloaded from Saccharomyces Genome Database (www.yeastgenome.org) in July 2011. These two files were named "2011.07.14_SGD_features.tab" and "2011.07.14_phenotype_data.tab," respectively.

Defining the set of yeast genes

To define our list of yeast genes using "2011.07.14_SGD_features.tab", non-protein coding genome features were removed (mitochondrial genes were retained). This list includes all features except for "ORF" (*not physically mapped, rRNA, autonomously replicating sequence, not in systematic sequence, centromere, external transcribed spacer region, 5' UTR, insertion, gene cassette, intron, long terminal repeat, +1 translational frameshift, pseudogene, repeat region, retrotransposon,*

telomere, telomeric repeat, transposable element gene, tRNA, snRNA, snoRNA, ncRNA, mating locus, multigene locus, non-transcribed region, noncodon exon, dubious, W region, X element combinatorial repeats, X region, Y region, Y' element, Z1 region, and Z2 region). Additionally, because many dubious ORFs overlap existing genes, their inclusion into our analysis would artificially duplicate yeast copy numbers of ortholog groups. Therefore, the list of 6,604 open reading frames was further refined by removal of the 806 dubious ORFs to define a set of 5,798 genes. This resulting list of genes can be found in the file "06-Results_Summary.txt."

Defining a set of yeast genes with unknown function

Among the 5,798 genes remaining are 4,931 that are listed in SGD as "verified" and 867 listed as "uncharacterized." For some of the analyses described in this manuscript, we were interested in defining a set of yeast genes whose biological role is unclear – genes with unknown function. To determine the total number of genes with unknown function in our gene set, we searched the SGD-provided gene descriptions of the "verified" ORFs for the phrase "unknown function." This resulted in 239 of 4,931 genes (each gene description was manually examined to confirm that the gene function was uncharacterized). Therefore, the list of 1,222 uncharacterized genes in our data set includes those annotated as uncharacterized (867 genes) and those that are verified but are described as having an unknown function (239 genes). This resulting designation for each gene can be found in the "Unknown Function" column of the file "06-Results_Summary.txt."

Assessment of gene deletion viability

The "2011.07.14_phenotype_data.tab" file was parsed by first removing all non-ORFs, as described above for the "SGD_features.tab" file. For our analysis, we were interested in whether or not a complete gene deletion has been annotated as inviable or viable (e.g. whether or not the gene is essential under normal growth conditions). We therefore only kept data for the "null" mutant type (*this included removal of the following mutant types: activation, conditional, dominant negative, gain of function, misexpression, overexpression, reduction of function, repressible, and unspecified*). Because null alleles have been engineered in multiple strain backgrounds, we opted for calling a gene essential if its deletion in any background resulted in inviability under normal growth conditions. For genes with an "inviable" null phenotype, we have included the genetic background information in parentheses. This resulting designation for each gene can be found in the "Null Phenotype" column of the file "06-Results_Summary.txt."

Among our defined set of yeast genes, we identified 4,250 gene deletions that are viable, 1,109 gene deletions that are inviable, and 325 gene deletions that have not been tested. The untested category consists mostly of genes that have been annotated after construction of the original systematic deletion collection, and also some genes encoded by the mitochondrial genome (GIAEVER *et al.* 2002). Interestingly, 114 genes in the untested category are present in the commercially available haploid deletion collection, suggesting that the gene deletion is viable under normal conditions (OpenBiosystems). A list of these genes is available in the file: "Untested_gene_deletions_in_haploid_collection.txt."

Data organization, processing, and visualization

Data were organized and processed using a combination of Microsoft Excel and R (www.r-project.org). Data was visualized using R or MultiExperiment Viewer (MeV_4_7, version 10.2; www.tm4.org/mev/). Both R and MultiExperiment Viewer are free, open-source software packages.

Identification of unreported gene deletions

In attempting to identify phenotypes (essential or non-essential) for the complement of protein-coding genes in yeast, we found a set of genes for which no gene deletion information is available in SGD (Saccharomyces Genome Database). This group of 325 genes contains genes that genuinely have not been published as deleted, but it also contains genes that are present in the haploid deletion collection from OpenBiosystems, and can be thus categorized as non-essential with as much confidence as any gene deletion in a large-scale collection. However, commercial availability does not constitute a curatable data source for SGD; a primary literature source is required (SGD personnel, personal communication). We have included this latter set of genes as downloadable data to provide such a data source.

File S2

Downloadable Data

Available as a zip file at http://www.g3journal.org/lookup/suppl/doi:10.1534/g3.113.006585/-/DC1
