# Supplementary material for: Phylogenetic Portrait of the Saccharomyces cerevisiae Functional Genome
Source: G3 (Bethesda). 2013 Aug 1;3(8):1335–40. doi: 10.1534/g3.113.006585 (PMC3737173; doi:10.1534/g3.113.006585)
Supplement: Supporting Information [file supp_g3.113.006585_FigureS1.pdf]

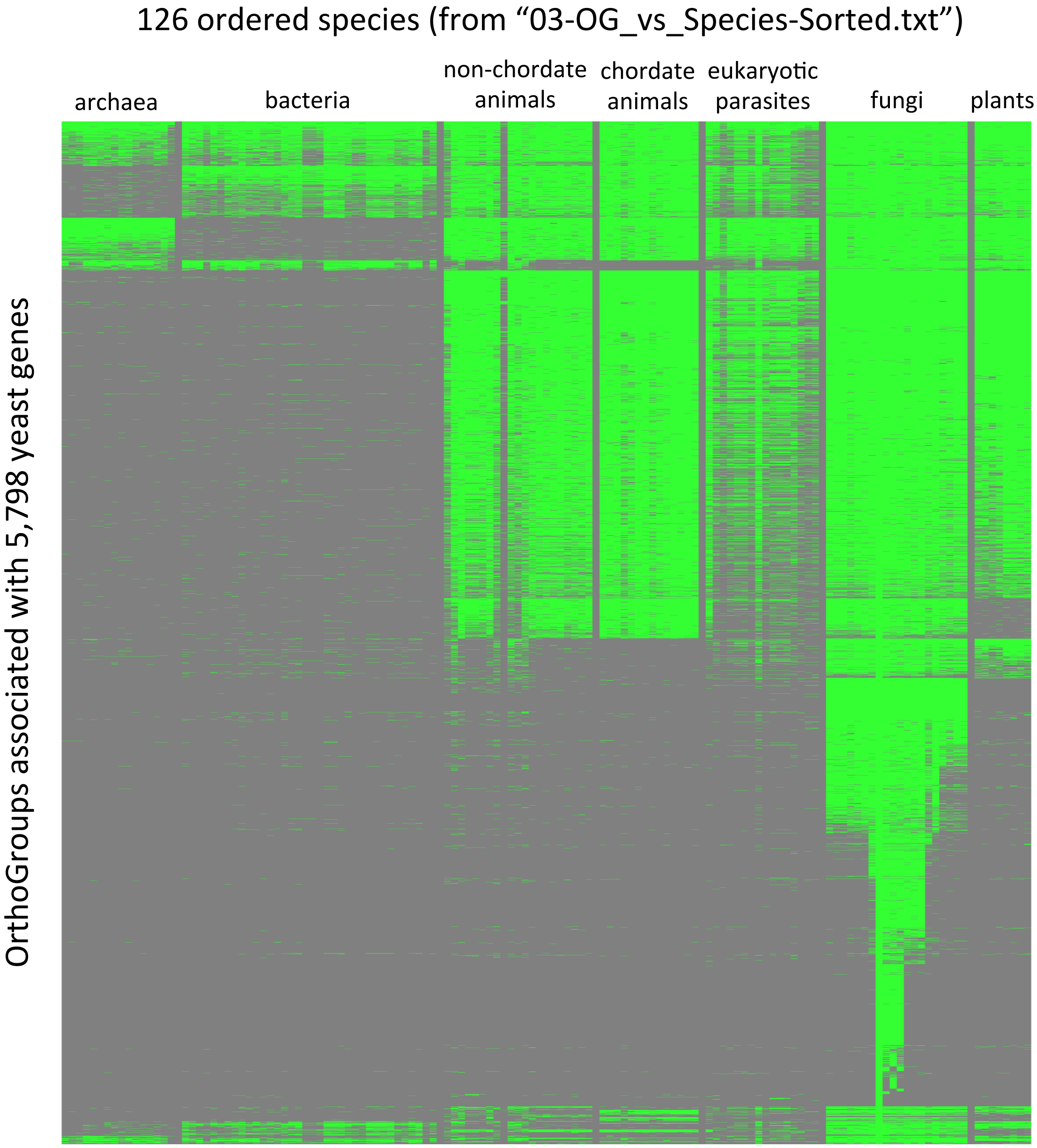


**Figure S1** Expanded heat-map showing conservation of yeast genes in each of the 131 species analyzed. Binarized data representing the presence or absence of an ortholog to each protein is represented as green (presence) or grey (absence) for each of the 126 species analyzed in this manuscript (a sub-set of the species present in the OrthoMCL database). The individual species data were collapsed into taxonomic groups for Figure 1. See the Materials and Methods section for details on data binarization, species selection, and ordering of genes.
