# Supplementary material for: Phylogenetic Portrait of the Saccharomyces cerevisiae Functional Genome
Source: G3 (Bethesda). 2013 Aug 1;3(8):1335–40. doi: 10.1534/g3.113.006585 (PMC3737173; doi:10.1534/g3.113.006585)
Supplement: Supporting Information [file supp_g3.113.006585_FigureS2.pdf]

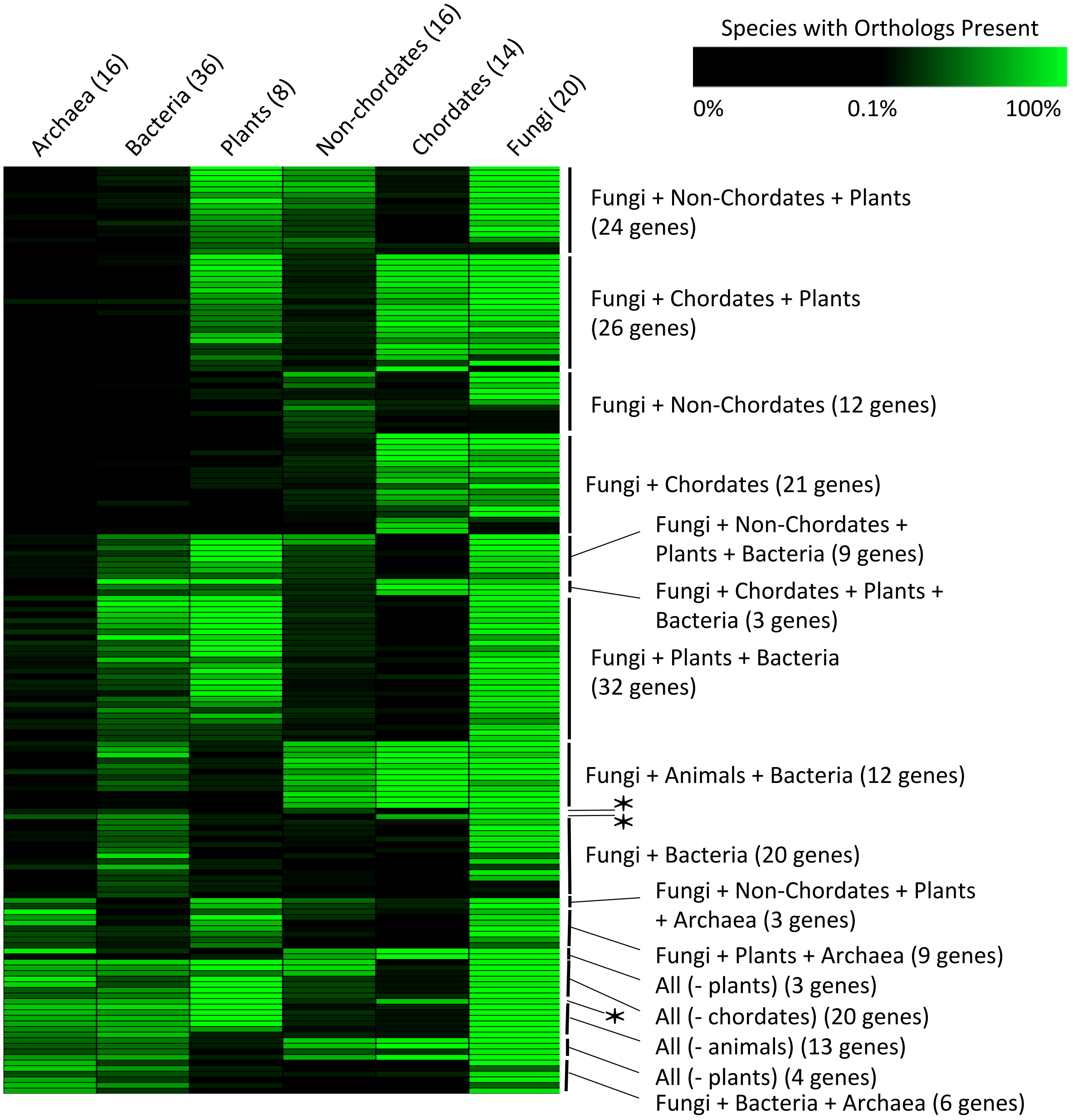

Figure S2 Fine-scale analysis of Minor Phylogroups. Expanded view of the Minor Phylogroups with included labels for rough phylogenetic categories to the right. An asterisk (*) indicates that only one gene is present with the identified phylogenetic pattern, and due to space limitations is not fully described in the phylogenetic categories to the right.
