# Supplementary material for: Phylogenetic Portrait of the Saccharomyces cerevisiae Functional Genome
Source: G3 (Bethesda). 2013 Aug 1;3(8):1335–40. doi: 10.1534/g3.113.006585 (PMC3737173; doi:10.1534/g3.113.006585)
Supplement: Supporting Information [file supp_g3.113.006585_FigureS3.pdf]

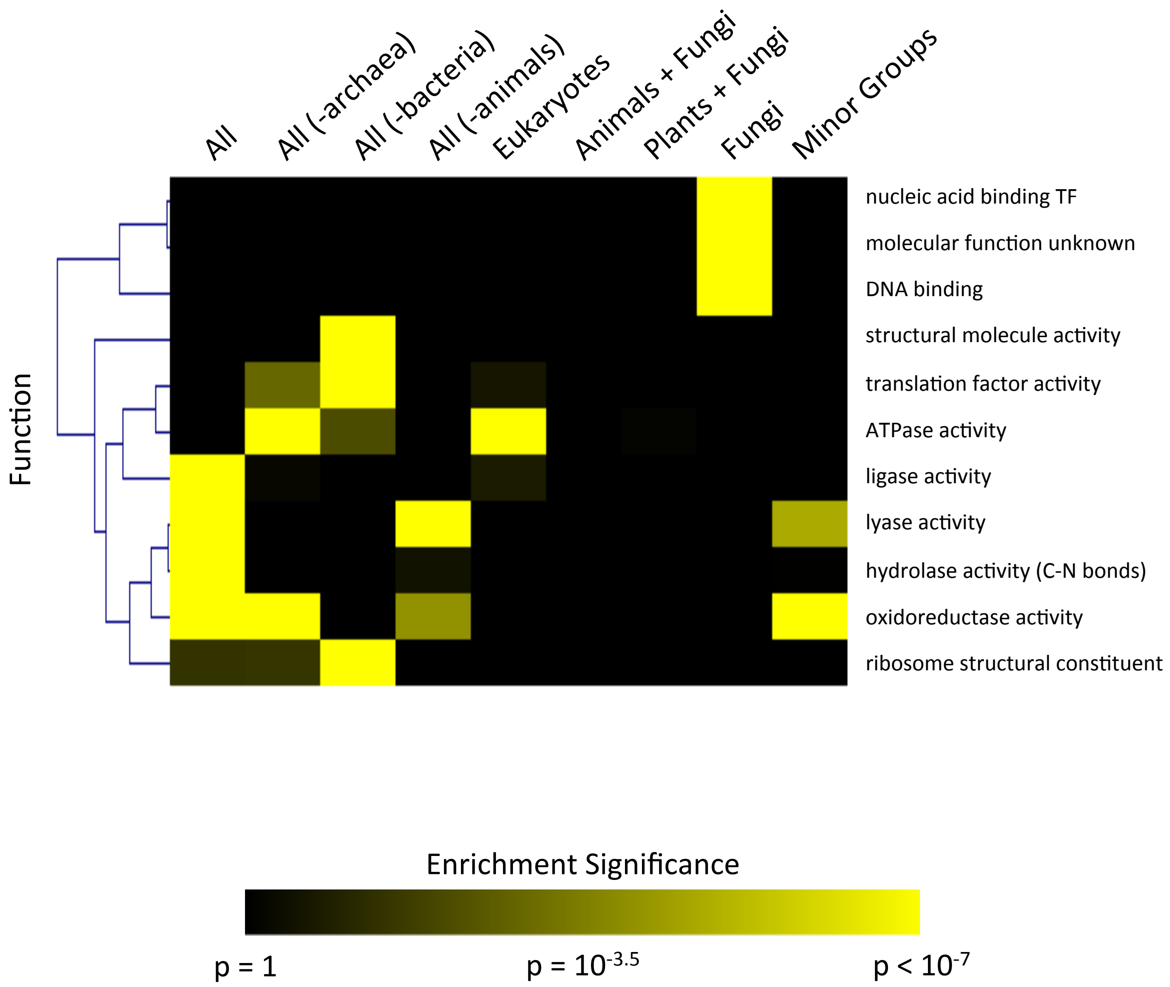

Figure S3 Gene Ontology (GO) functional category term enrichment of phylogroups. GO-Slim Mapper was used to identify GO terms that are enriched in each phylgroup. The most significant results are presented in a heat-map with yellow intensity corresponding to significance of enrichment (see legend - the color intensity scale was defined using our significance threshold of $p < 10^{-7}$). Phylogroups analyzed are listed across the top of the heat-map.
