# Supplementary material for: Phylogenetic Portrait of the Saccharomyces cerevisiae Functional Genome
Source: G3 (Bethesda). 2013 Aug 1;3(8):1335–40. doi: 10.1534/g3.113.006585 (PMC3737173; doi:10.1534/g3.113.006585)
Supplement: Supporting Information [file supp_g3.113.006585_FigureS5.pdf]

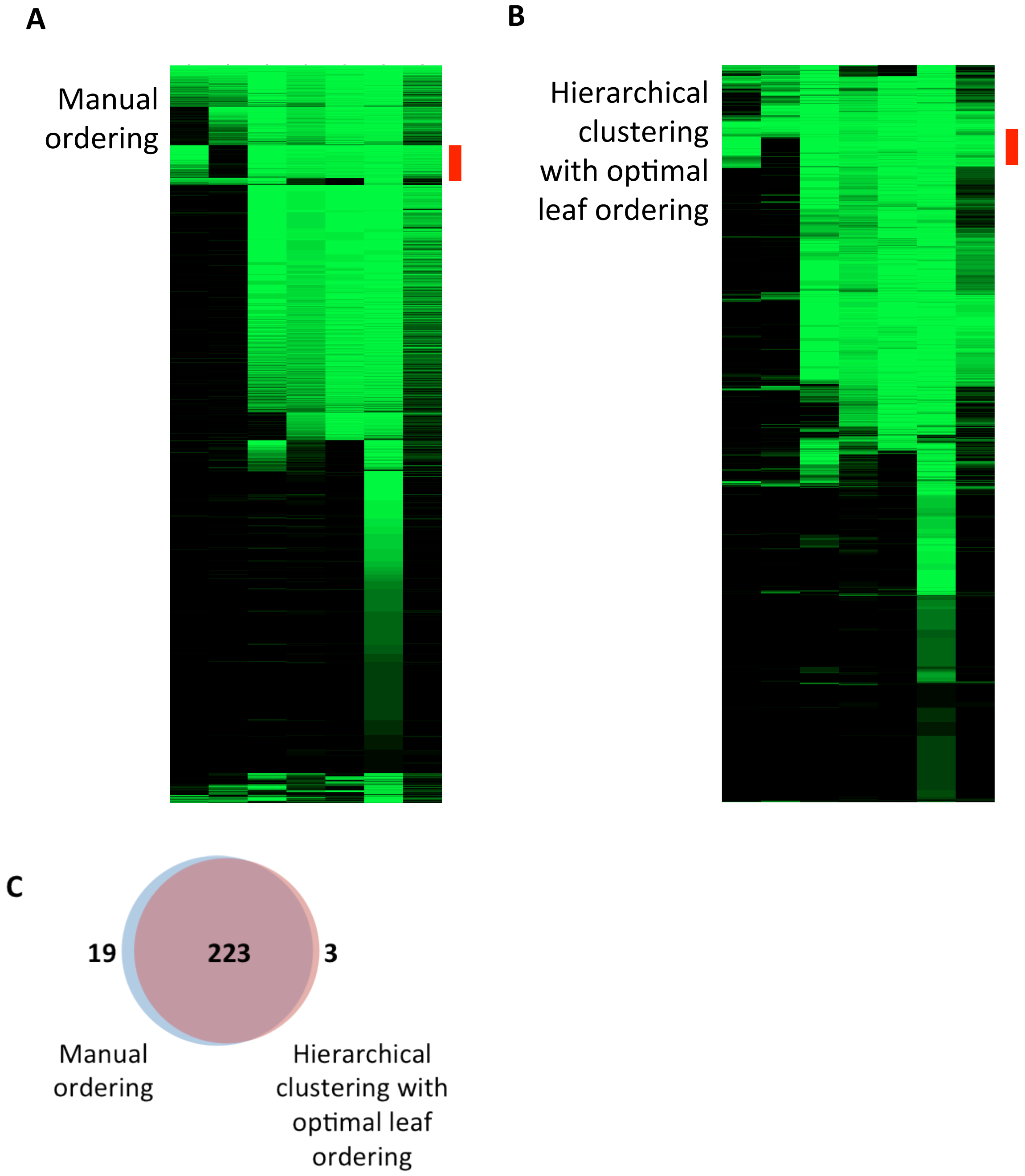
A
Manual ordering
B
Hierarchical clustering with optimal leaf ordering
C
19
223
3
Manual ordering
Hierarchical clustering with optimal leaf ordering

Figure S5 Alternative clustering approaches result in similar clusters of genes. Comparison of manual ordering (employed in this study) and hierarchical clustering with optimal leaf ordering as in Bar-Joseph, 2001 (note that in accordance with the original figure the data for panel B was binarized using the 0.2 threshold and eukaryotic parasites were not included for the clustering). (A) and (B) The column order is the same as in Figure 1. The red bar refers to the group of genes found in all species except bacteria. Note that the main difference appears to be in the scattering of genes that were placed into a group called "minor clusters" for the original figure. (C) Venn diagram showing overlap of the genes (in all species except bacteria) identified by each method. The overlap is highly significant ($p<10^{-308}$, hypergeometric distribution).
