# Supplementary material for: Phylogenetic Portrait of the Saccharomyces cerevisiae Functional Genome
Source: G3 (Bethesda). 2013 Aug 1;3(8):1335–40. doi: 10.1534/g3.113.006585 (PMC3737173; doi:10.1534/g3.113.006585)
Supplement: Supporting Information [file supp_g3.113.006585_FileS2.zip › Downloadable Data/README.doc]

**This file is intended to explain the contents of each file available for download. Provided file names are shown in red, followed by a description of the contents in italicized text.**

**------------------------------------------------------------------------------------------------------------------------**

2011.07.14_phenotype_data.txt

2011.07.14_SGD_features.txt

These files contain the raw data downloaded from the *Saccharomyces cerevisiae* Database (SGD; www.yeastgenome.org). The files were downloaded on July 14, 2011. The files use the same column headings described by SGD.

Classification_of_OrthoMCL_Species.txt

This file contains each organism that the OrthoMCL database has curated, followed by a taxonomic break-down of each used to group organisms together.

Column headings (*and descriptions*) are as follows:

Organism (*Genus and species name (and strain information) provided by OrthoMCL*)

OrthoMCL Number (*Organism number assigned by OrthoMCL*)

OrthoMCL Abbreviation (*4-letter organism abbreviation used by OrthoMCL*)

\# Sequences Analyzed (*Number of ORF sequences analyzed by OrthoMCL*)

\# Sequences Clustered (*Number of ORF sequences clustered by OrthoMCL*)

\# Groups (*Number of OrthoGroups into which clustered ORFs were sorted*)

Domain, Kingdom, Subkingdom, etc. (*taxonomic descriptions of each organism*)

01-OG_vs_Species-Full.txt

This file contains a matrix with the number of orthologs in each species present in the OrthoMCL database. The columns represent each species (listed in first row). The rows represent OrthoMCL groups (the only groups analyzed in this data are those present in *S. cerevisiae*).

02-OG_vs_Species-Binary.txt

This file is a transformed version of the above file. All data regarding numbers of present orthologs per species was reduced to either “0” for no orthologs present, or “1” for at least one ortholog present.

03-OG_vs_Species-Sorted.txt

This file contains the same information presented in file 02; however, some of the species have been removed (see Methods section for more details) and all have been re-ordered into groups of taxonomically similar organisms.

04-OG_vs_Groups.txt

This file was generated by collapsing data from individual species into groups. For example, if a particular ortholog was present in 12/16 archaeal species examined, a value of 0.75 is entered for that OrthoGroup in the “Archaea” column.

05-Ordered_PhyloGroups.txt

This file contains the data above merged with information that maps each OrthoGroup to a *S. cerevisiae* gene. The data is ordered based on manual clustering as described in the Methods section.

Column headings (*and descriptions*) are as follows:

Clustered Order (*the order of each gene based on manual clustering*)

Feature Name (*S. cerevisiae Open Reading Frame (ORF) designation*)

OrthoGroup (*Orthology Group number assigned by OrthoMCL database*)

PhyloGroup (*Phylogenetic grouping based on our analysis; number and description listed*)

… each organism group defined in file 04 constitute the remaining columns.

06-Results_Summary.txt

This file contains the data above merged with individual gene phenotypes, names, and descriptions (information from Saccharomyces Genome Database files outlined above).

Column headings (*and descriptions*) are as follows:

SGDID (*SGD identification code for an individual ORF*)

Feature Qualifier (*SGD characterization of an ORF as either “verified” or “uncharacterized”*)

Feature Name (*S. cerevisiae Open Reading Frame (ORF) designation*)

OrthoGroup (*Orthology Group number assigned by OrthoMCL database*)

PhyloGroup (*Phylogenetic grouping based on our analysis; number and description listed*)

PhyloGroup Clustered Order (*the order of each gene based on manual clustering*)

Null Phenotype *(lists whether the gene deletion is “viable” or “inviable;” if “inviable,” strains*

*are listed where this result was shown)*

Unknown Function *(lists whether or not this gene is considered to have an “unknown*

*function” for the purposes of our analysis. See Methods section for details.)*

Gene Name *(Primary assigned gene name in SGD; ORF designation if no gene name assigned)*

Description *(Description of gene function provided by SGD)*

GO-Slim_Analysis_full.txt

Contains the results from Gene Ontology GO-Slim searches performed using the GO-Slim analysis tool on SGD (searches were performed in September 2011). GO-Slim searches were performed for groups of genes present in PhyloGroups 0-8.

Column headings (*and descriptions*) are as follows:

PhyloGroup (*PhyloGroup number*)

GO-Category (*Either “Component,” “Function,” or “Process”)*

GOID (*GO-term identification number*)

GO term (*gene ontology term defined as part of the GO-Slim set*)

No. in PhyloGroup (*Number of GO term associated genes for defined PhyloGroup*)

PhyloGroup Total (*Number of genes in defined PhyloGroup*)

No. in Genome (*Number of GO term associated genes for defined PhyloGroup in genome*)

Genome (*Total number of genes in genome – designated by the GO-Slim tool*)

P-value (hygecdf) (*p-value calculated using cumulative hypergeometric distribution*)

P-value (*BY (Benjamini-Yekutieli) multiple-hypothesis corrected p-value*)

PhyloGroup v. Genome (*lists whether the GO-term gene set in each PhyloGroup is either*

*“underrepresented” or “enriched” compared to the same set in the genome*)

log2(fold-change) (*log2-transformation of fold-change comparing phylogroup to entire*

*genome, as follows (log2(fraction in phylogroup)/(fraction in genome))*)

Gene(s) (*lists genes present in intersection of GO-Slim term and PhyloGroup*)

Untested_gene_deletions_in_haploid_collection.txt

This file contains the 114 genes listed in SGD as not having a deletion phenotype, but are also present in the OpenBiosystems haploid deletion collection.
